# Environmental filtering shapes biosynthetic potential and resistome of antarctic microbiomes

**DOI:** 10.1007/s11274-026-05201-8

**Published:** 2026-08-14

**Authors:** William B. Medeiros, Kelly J. Hidalgo-Martinez, Daniel D. P. S. Penna, Valéria M. Oliveira

**Affiliations:** 1 Microbial Resources Division, Research Center for Chemistry, Biology, and Agriculture (CPQBA), Universidade Estadual de Campinas (UNICAMP), Paulínia, São Paulo Brazil; 2 Biology Institute, Universidade Estadual de Campinas – UNICAMP, Campinas, São Paulo Brazil; 3 Synthetic Biology Laboratory, Department of Structural and Functional Biology, Universidade Estadual de Campinas – UNICAMP, Campinas, Brazil

**Keywords:** Biosynthetic Gene Clusters, Microbial Resistome, Environmental Filtering, Adaptive Specialization, Extreme Environment

## Abstract

Environmental filtering is a major driver of microbial community assembly in Antarctic ecosystems, yet its influence on biosynthetic potential and antimicrobial resistance remains poorly understood. Here, we analyzed 319 medium- to high-quality metagenome-assembled genomes (MAGs) recovered from four Antarctic sites (Whalers Bay, Crater Lake, Fumarole Bay, and Hannah Point) to investigate the relationship between geochemical gradients, biosynthetic gene clusters (BGCs), and antimicrobial resistance genes (ARGs). Integrating genome-resolved metagenomics, biosynthetic mining, resistome profiling, and environmental analyses, we identified 1,197 BGCs, with terpene clusters representing more than 25% of the total. Several biosynthetic hotspots were detected, including an Acidobacteriota MAG harboring 62 BGCs. Resistome composition exhibited strong site-specific structuring and was significantly associated with geochemical variables, particularly cobalt, iron, organic carbon, and thermal variation. Network analyses revealed highly connected MAGs affiliated with Pseudomonadota and Actinomycetota, linking diverse BGC and ARG classes. At the same time, genomic co-localization of biosynthetic and resistance determinants suggests potential adaptive associations between secondary metabolism and self-resistance mechanisms. Together, these findings demonstrate that environmental filtering shapes both the taxonomic and functional organization of Antarctic microbiomes and highlight polar ecosystems as reservoirs of unexplored biosynthetic diversity with potential biotechnological relevance.

**Supplementary Information:**

The online version contains supplementary material available at https://doi.org/10.1007/s11274-026-05201-8.

## Introduction

Antarctic bacteria exhibit unique physiological and metabolic traits that diverge significantly from those in temperate environments, reflecting millions of years of evolution under extreme selection pressures. Survival in these habitats requires specialized adaptations to freezing temperatures, intense ultraviolet radiation, and nutrient scarcity, which have led to the evolution of cold-active enzymes, cryoprotectants, and highly efficient DNA repair mechanisms (Ramasamy et al. 2023). Beyond physiological resilience, the geographical isolation of the Antarctic continent has fostered distinct microbial communities, with studies revealing that the Antarctic and Arctic poles share only approximately 30% of their microbial taxa (Kleinteich et al. 2017). This isolation, combined with site-specific selection, has endowed these microorganisms with a prolific biosynthetic capacity. Indeed, Antarctic microbes harbor a rich diversity of biosynthetic gene clusters (BGCs) encoding specialized metabolites, ranging from pigments to novel antibiotics, including terpenes, polyketides (PKs), non-ribosomal peptides (NRPs), and ribosomally synthesized and post-translationally modified peptides (RiPPs) (Waschulin et al. 2021; Benaud et al. 2020; Medeiros et al. 2024; Hwengwere et al. 2025).

In parallel with this biosynthetic diversity, Antarctic environments also serve as reservoirs of antimicrobial resistance (AMR), where resistance profiles are shaped by both ancestral evolution and contemporary anthropogenic influences (Jara et al. 2020; Bisaccia et al. 2025; Hwengwere et al. 2022). Notably, resistance mechanisms, particularly multi-drug efflux pumps, are widely distributed across both culturable and unculturable taxa and target diverse antibiotic classes, including β-lactams, tetracyclines, and quinolones (Silverio et al. 2025; Marcoleta et al. 2021; Wang et al. 2022). While human activity near research stations has been linked to a high prevalence of Antimicrobial Resistance Genes (ARGs), pristine Antarctic soils also harbor ancient, uncharacterized ARGs that represent a potential source of novel clinical mechanisms (Marcoleta et al. 2021; Na et al. 2020; Dimov and Strateva 2022). The co-occurrence of BGCs and ARGs within a single genome is not coincidental; this proximity allows producers of antimicrobial compounds to survive their own bioactive products while gaining a competitive advantage in resource-limited niches (Busi et al. 2021; Xiao et al. 2023; Núñez-Montero et al. 2022). This genomic relationship likely influences microbial competition and community dynamics, yet the environmental factors that dictate the spatial organization and distribution of these functional “hubs” remain poorly understood.

Despite growing evidence of biosynthetic diversity and antimicrobial resistance in Antarctic microbes, how environmental pressures structure these functional traits at the genome level remains poorly understood. Here, we sought to determine how geochemical heterogeneity influences the distribution of BGCs and ARGs in Antarctic microbiomes. To address this question, we analyzed microbial communities inhabiting contrasting geochemical environments across Deception and Livingston Islands in the Antarctic Peninsula using genome-resolved metagenomics integrated with biosynthetic and resistance profiling. By examining the relationship between geochemical gradients and microbial functional traits, this study aims to clarify how environmental stress shapes the genomic architecture of specialized metabolism and antimicrobial resistance in polar ecosystems.

## Materials and methods

### Environmental settings, datasets, and MAG recovery

This study builds on a previously published, publicly available dataset that recovered metagenome-assembled genomes (MAGs) from Antarctic soil metagenomes. Here, we provide a summary of the MAG recovery pipeline for completeness, while detailed descriptions of sampling, DNA extraction, library preparation, and sequencing are reported in the original data publication (Medeiros et al. 2026). Briefly, raw metagenomic sequencing data from Deception Island (Crater Lake, − 62.9811, − 60.6675; Fumarole Bay, − 62.9674, − 60.7099) and Livingston Island (Hannah Point, − 62.6470, − 60.6031) were quality filtered using Trimmomatic v0.39 (Bolger et al., 2014) to remove low-quality bases (Phred score ≤ 30). High-quality reads were assembled into contigs using metaSPAdes v3.15.5 (Nurk et al. 2017). To support genome binning, sequencing reads were mapped back to assembled contigs using Bowtie2 v2.2.6 (Langmead and Salzberg 2012), and depth information was generated using samtools v0.1.19 (Li et al. 2009). Five complementary binning approaches were applied: CONCOCT v1.1 (Alneberg et al. 2014), MaxBin2 v2.2.6 (Wu et al. 2016), SemiBin2 (Pan et al. 2023), MetaCoAG (Mallawaarachchi andLin 2022), and MetaBAT2 v2.12 (Kang et al. 2019). The resulting bins were dereplicated using MAGScoT (Rühlemann et al. 2022). MAG quality was assessed using CheckM v1.1.2 (Parks et al. 2015), and only genomes with ≥ 50% completeness and ≤ 10% contamination were retained for downstream analyses. In addition to the MAGs recovered from Crater Lake, Fumarole Bay, and Hannah Point, we included 180 MAGs previously reconstructed from Whalers Bay (− 62.9791, − 60.5556) (Centurion et al. 2022), another site on Deception Island. These genomes are also part of the publicly available dataset reported in Medeiros et al. (2026), enabling comparative analyses across all four Antarctic sites.

### Taxonomic assignment, phylogeny, and functional annotation of MAGs

Taxonomic classification and phylogenetic inference were performed using GTDB-Tk (Chaumeil et al. 2019) based on the Genome Taxonomy Database (GTDB) release R207. Functional potential was assessed by screening all MAGs for BGCs using antiSMASH v7.0 (Blin et al. 2023) with default parameters. Putative biological activities of predicted BGCs were inferred using NPBDetect, a machine learning–based tool trained on MIBiG-annotated biosynthetic gene clusters (Goyat et al. 2025). Open reading frames were predicted with Prodigal v2.6.3 (Hyatt et al. 2010), and the resulting protein sequences were searched against the latest release of the Comprehensive Antibiotic Resistance Database (CARD v4.0.1) (Alcock et al. 2022) using DIAMOND v2.1.8 (coverage identity 60) (Buchfink et al. 2021) in BLASTp mode. ARGs were classified into AMR categories and resistance mechanisms according to the CARD ontology, and abundance was normalized as reads per kilobase per million mapped reads (RPKM) using an in-house R pipeline.

Data manipulation and visualization were conducted in R v4.5.1 using the tidyverse (Wickham et al. 2019) and ggplot2 (Wickham 2016) packages. A phylogenetic tree was constructed in iTOL (Letunic and Bork 2024) to depict evolutionary relationships and was annotated with branch lengths and taxonomic classifications. ARG distribution by antimicrobial resistance mechanism was represented in a radial layout using RAWGraphs (Mauri et al. 2017).

### Resistome structure and comparative analysis

To investigate the spatial distribution of the resistome across the four sampling sites, we employed a multidimensional statistical approach using the vegan (v2.6-4) and pheatmap (v1.0.12) packages in R. Initially, an abundance matrix of ARGs was consolidated, and a distance matrix was calculated based on the Bray-Curtis dissimilarity index. Resistome β-diversity was visualized using Principal Coordinates Analysis (PCoA). To accommodate the uneven sample sizes, group cohesion was represented using centroid-based segments (spider plots). The statistical significance of the observed clustering was validated using Permutational Multivariate Analysis of Variance (PERMANOVA) via the adonis2 function. We performed 999 permutations to assess the influence of the sampling site on the resistome profile. The magnitude of the site effect was quantified using the coefficient of determination (R^2^). Pairwise PERMANOVA comparisons were conducted with Bonferroni correction to control for Type I error across multiple comparisons.

To identify the specific genes driving the differentiation between sites, a Similarity Percentages (SIMPER) analysis was performed, which decomposes Bray-Curtis dissimilarities into the percentage contributions of individual ARGs. The top 10 genes contributing to the majority of dissimilarity (cumulative contribution > 80%; Supplementary Table 1) were selected for further visualization. Finally, a hierarchical clustering heatmap was generated using Z-score-normalized abundances to illustrate the relative enrichment of these key ARGs across the sampled sites, thereby facilitating correlations between specific resistance profiles and their geographic origins.

### Environmental drivers of the resistome structure

To investigate the relationship between environmental factors and the resistome structure, we performed a distance-based Redundancy Analysis (dbRDA) using Bray-Curtis distance. This constrained ordination technique was employed to quantify the extent to which the observed β-diversity could be explained by the soil’s physicochemical properties (metals, nutrients, carbon, and temperature).

Before the dbRDA, a multicollinearity assessment was conducted to ensure that the environmental predictors were independent. We calculated the Variance Inflation Factor (VIF) for all fifteen initial variables (Supplementary Table 2). To prevent model overparameterization and biased coefficient estimates, variables were stepwise excluded based on a VIF threshold of > 5. The remaining eight variables (Cobalt [Co], Iron [Fe], Lead [Pb], Organic Carbon [Corg], Sediment Temperature [TempSe], Total Nitrogen [N_total], Ammonium [NH4+], and Nitrate [NO3−]; Supplementary Table 3) represented independent environmental gradients.

The final dbRDA model was constructed using the dbrda function from the vegan package, with the Bray-Curtis distance matrix as the response variable and the VIF-filtered environmental parameters as explanatory variables. The global significance of the model was assessed using a permutation test (999 permutations), and the goodness of fit was quantified with the adjusted coefficient of determination (R^2^_adj_) to account for the number of predictors. Furthermore, the marginal significance of each environmental variable was evaluated using a permutation test per term (by = “terms”) to identify the specific drivers significantly shaping the resistance gene profiles (*p* < 0.05). All results were visualized in a dbRDA triplot, in which environmental vectors were projected onto the ordination space, and the length and direction of the arrows indicated their relative influence. Finally, to identify the biological signatures associated with the identified environmental drivers, taxonomic relative abundance was calculated at the Phylum level. To visualize site-specific partitioning, a hierarchical clustering analysis was performed on a transposed relative abundance matrix. Samples were clustered using Ward’s D2 method (Ward 1963) based on Euclidean distances to evaluate the congruence between taxonomic assemblages and the site-specific clusters observed in the dbRDA ordination. A heatmap was generated to illustrate the relative enrichment of specific phyla across environmental gradients, with zero-abundance values explicitly identified to highlight presence-absence patterns across contrasting sites.

### Tripartite MAG–ARG–BGC networks

To investigate the integration between taxonomic identity, antibiotic resistance, and secondary metabolite biosynthesis, we built a tripartite MAG–ARG–BGC network using the curated annotation table. Each MAG was collapsed to a single row containing presence/absence profiles for all detected ARG classes and BGC categories. These presence/absence matrices were transformed into two bipartite edge sets (MAG–ARG and MAG–BGC), which were subsequently merged into a unified tripartite graph. Network construction and centrality calculations were performed using the igraph (Csárdi and Nepusz 2006) and tidygraph packages, while visualization was conducted with ggraph (Pedersen, 2025). Rare classes (degree < 3) were removed to reduce noise and improve interpretability, MAGs without any functional annotations (BGC and/or ARG) were excluded, and the analysis was restricted to the most abundant phyla. The network was visualized using a Kamada–Kawai layout to separate functional clusters while preserving local connectivity, and centrality metrics were used to identify influential MAGs, ARG classes, and BGC types.

## Results

**Fig. 1:**
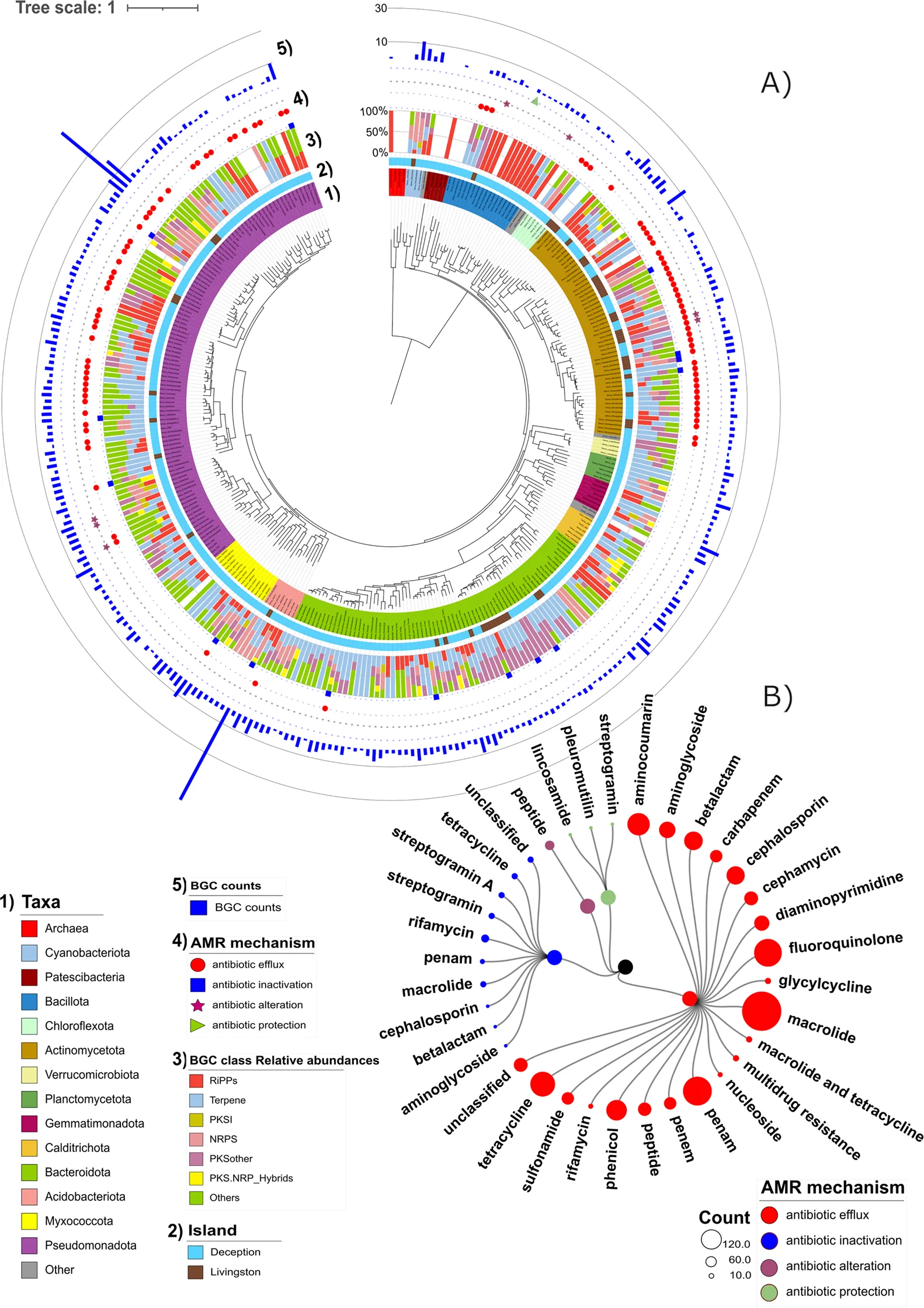
Circular phylogenomic tree of 319 metagenome-assembled genomes (MAGs) recovered from Deception and Livingston Islands, Antarctica, showing (**A**) the distribution of biosynthetic gene clusters (BGCs) and antimicrobial resistance (AMR) mechanisms. The innermost ring (1) indicates taxonomic classification at the phylum level, followed by (2) island origin, (3) relative abundances of BGC classes, (4) AMR mechanisms categorized by resistance strategy, and (5) total BGC counts per MAG. The network at the bottom-right (**B**) shows the diversity and frequency of ARGs and their associated resistance mechanisms

We analyzed 319 medium- to high-quality MAGs from four Antarctic sites: Whalers Bay, Crater Lake, and Fumarole Bay on Deception Island, and Hannah Point on Livingston Island. Taxonomic characterization revealed microbial diversity, with the recovered genomes spanning 19 distinct bacterial phyla. This broad phylogenetic distribution highlights the ecological complexity and biosynthetic potential reflected in the reconstructed genomes (Fig. 1). The dominant phyla were Pseudomonadota, Bacteroidota, and Actinomycetota, followed by less-characterized phyla such as Verrucomicrobiota, Patescibacteria, and Calditrichota (Fig. 1A). Among the broadly distributed BGC classes, Terpenes and RiPPs were the most common across phyla. On average, approximately six BGCs were detected per MAG. However, one MAG, classified as Acidobacteria (*CL_7_Thermoanaerobaculia*), stood out by harboring an exceptionally high number of BGCs (*n* = 62), including 42 nonribosomal peptide synthase (NRPS) clusters (Fig. 1A).

The outer ring number 4 of the tree (Fig. 1A) reveals considerable overlap between taxa enriched in secondary metabolite production (e.g., NRPS, PKSI) and those exhibiting AMR mechanisms, notably antibiotic efflux pumps (marked by red circles) and antibiotic inactivation (blue squares). The antibiotic efflux pump mechanism was particularly prevalent, especially within Pseudomonadota and Actinomycetota. Resistance genes associated with AMR mechanisms, such as those conferring resistance to macrolide, fluoroquinolone, and tetracycline antibiotics, were the most abundant (Fig. 1B).

### Biosynthetic diversity and distribution

**Fig. 2:**
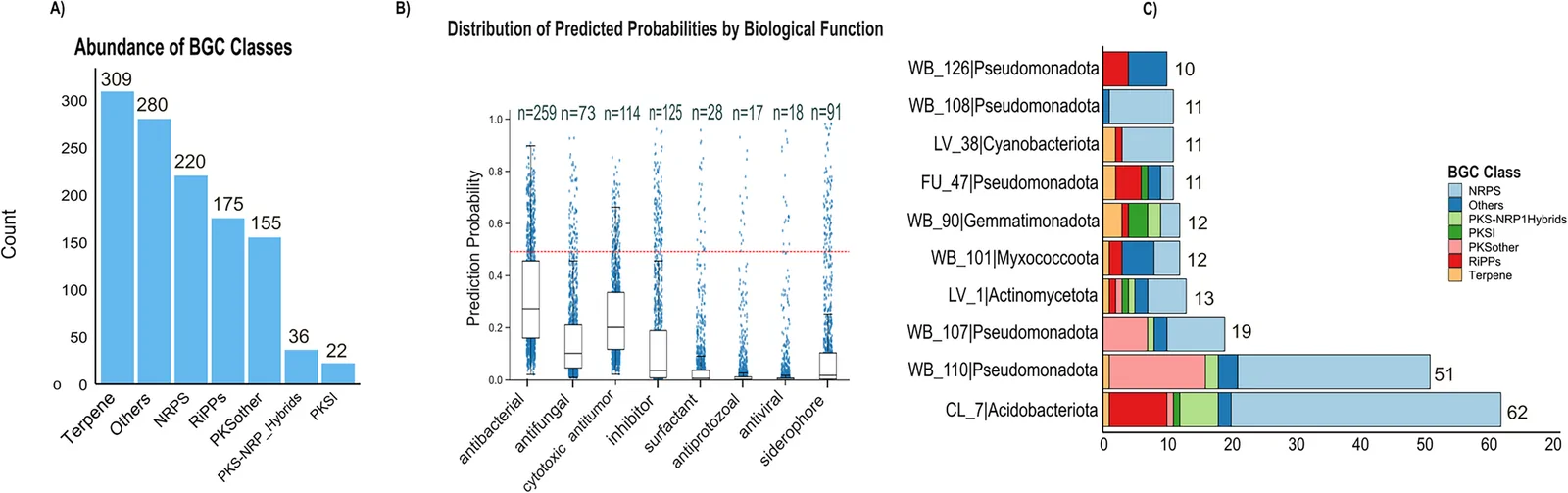
Functional Potential of Biosynthetic Gene Clusters. (**A**) Bar chart showing the total count of BGCs identified across the dataset, categorized by class. (**B**) Distribution of predicted probabilities for eight biological functions. Data points above the red dashed line represent BGCs with high-confidence functional assignments. (**C**) Taxonomic breakdown of BGC classes among the top 10 most prolific MAGs. The length of the stacked bars represents the total BGC count per MAG, with colors corresponding to specific biosynthetic classes

Metagenome mining for BGCs in the recovered MAGs revealed biosynthetic potential within Antarctic microbial communities. A total of 1,197 BGCs were identified and classified (Fig. 2A). Terpene clusters represented the most abundant class (*n* = 309), accounting for over 25% of the total BGC content, followed by NRPS (*n* = 220) and RiPPs (*n* = 175). Polyketide synthase-related clusters (including PKS-other, PKS-NRPS hybrids, and PKSI) were less frequent but remained significant, totaling 213 clusters.

To assess the biotechnological potential of the identified BGCs, we employed NPBDetect, a neural network model that predicts natural product bioactivity from BGCs identified by antiSMASH. This tool enabled screening of eight distinct functional categories: antibacterial, antifungal, antitumor/cytotoxic, siderophore, antiprotozoal, inhibitor, antiviral, and surfactant. The distribution of predicted probabilities by biological function is illustrated in Fig. 2B. The boxplots illustrate the functional potential inferred from the reconstructed genomes, with values above the dashed line indicating BGCs predicted to encode biological functions with statistical confidence. A substantial number of BGCs reached this threshold across several categories, including those of high global relevance to the AMR crisis. Specifically, the antibacterial (259) and antifungal (73) categories exhibited a high probability of encoding antimicrobial functions. The taxonomic distribution of these clusters highlighted specific “hotspots” of biosynthetic diversity among the ten most enriched MAGs (Fig. 2C). Notably, MAGs WB_110 (Pseudomonadota) and CL_7 (Acidobacteriota) exhibited the highest BGC counts, harboring 51 and 62 clusters, respectively, with a primary dominance of NRPS. Moreover, while WB_110 showed a high proportion of PKS-other, CL_7 displayed a more heterogeneous profile, including RiPPs and hybrid clusters. Moderate BGC counts (12–13 clusters) were observed in LV_1 (Actinomycota), WB_101 (Myxococcota), and WB_90 (Gemmatimonadota).

Site-specific analysis further revealed that BGC profiles within individual phyla shifted according to geographical location (Supplementary Fig. 1). The Bacteroidota transitioned from a terpene- and PKS-predominant profile at Crater Lake to a more diverse repertoire at Fumarole Bay and Whalers Bay. Similarly, Actinomycetota was restricted to RiPP BGCs at Fumarole Bay but exhibited high diversity of BGC classes at other sampled sites. The most prolific phyla varied by site: Acidobacteriota and Pseudomonadota at Crater Lake; Bacteroidota, Planctomycetota, and Pseudomonadota at Fumarole Bay; and a broader set including Gemmatimonadota and Verrucomicrobiota at Whalers Bay.

### Spatial structuring of the antarctic resistome

**Fig. 3:**
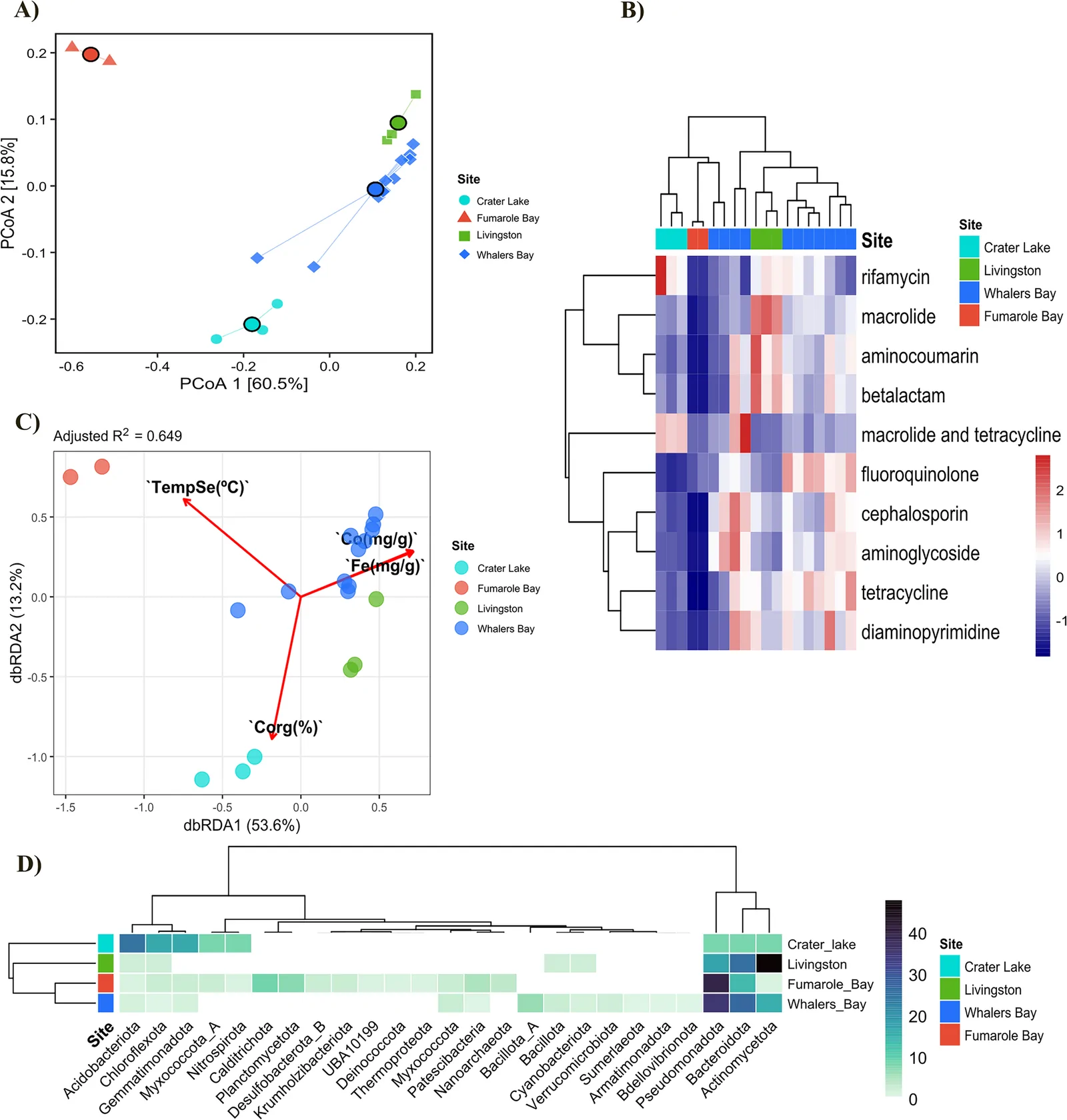
Resistome Structure and Environmental Drivers. (**A**) PCoA of ARG β-diversity based on Bray-Curtis dissimilarity, with centroid-based segments illustrating site-specific clustering. (**B**) Hierarchical Clustered Heatmap of Z-score normalized abundances for the top 10 “driver” ARGs identified via SIMPER analysis. (**C**) dbRDA triplot (R²_adj_ = 0.649) showing the influence of independent environmental vectors (red arrows) on resistome structure. (**D**) Taxonomic Heatmap at the Phylum level clustered by Ward’s D2 method, illustrating niche-specific partitioning and lineage enrichment across sites

Resistome composition was strongly structured by sampling site. PCoA revealed a clear separation of ARG profiles according to geographic location (Fig. 3A). This pattern was statistically supported by PERMANOVA (Supplementary Table 4), which showed that sampling site explained 79.2% of the total variance in ARG composition (R² = 0.792, F = 19.102, *p* = 0.001). Pairwise comparisons (Supplementary Table 5) further resolved site-specific differences. Whalers Bay exhibited a resistance profile significantly distinct from both Crater Lake (*p* = 0.024) and Livingston (*p* = 0.018). In contrast, comparisons involving Fumarole Bay versus Crater Lake and Fumarole Bay versus Livingston produced high R² values (0.89–0.95), indicating strong biological divergence, but did not reach statistical significance (*p* = 0.600), likely reflecting the limited sample size (*n* = 2–3), a common constraint in studies of remote Antarctic environments where sample collection is logistically challenging.

To identify the main contributors to this spatial differentiation, SIMPER analysis was applied. A small subset of ARGs collectively accounted for 84.9% of the total dissimilarity among sites (Supplementary Table 6, top 10 ARGs), with genes conferring resistance to macrolide, tetracycline, and aminocoumarin antibiotics emerging as the dominant contributors. Macrolide resistance genes were notably enriched in Whalers Bay (mean abundance: 147.1) compared to Crater Lake (mean abundance: 45.8). Additionally, specific ARGs, including rifamycin resistance and macrolide/tetracycline co-resistance genes, were detected exclusively in Whalers Bay. Hierarchical clustering of the top 10 contributing ARGs (Z-score-normalized) reinforced these patterns (Fig. 3B), highlighting the distinct resistome signature of Whalers Bay, characterized by higher ARG abundance and diversity, which are largely absent or reduced at other sites.

### Environmental drivers of resistome composition

Environmental gradients explained a substantial fraction of the observed resistome variability (Fig. 3C). The dbRDA, performed after excluding highly collinear variables (VIF > 5), retained eight independent environmental predictors (Supplementary Table 3) and significantly accounted for 64.8% of the total variation in ARG composition (adjusted R² = 0.648; permutation test).

Marginal tests identified cobalt concentration (F = 14.52; *p* = 0.001) and sediment temperature (F = 8.52; *p* = 0.003) as the strongest individual predictors. Iron (*p* = 0.006) and organic carbon (*p* = 0.015) also contributed significantly to resistome structuring. In contrast, lead (*p* = 0.057) and total nitrogen (*p* = 0.082) showed only marginal influence, and inorganic nitrogen species (ammonium and nitrate) were not significantly associated with ARG distribution (Table 1).

**Table 1:** Results from distance-based redundancy analysis (dbRDA) identifying the individual contribution of heavy metal concentrations, sediment temperature, and nutrient profiles to the resistome structure. Significance was determined using permutation tests (*n* = 999). Df = degrees of freedom; F = pseudo-F statistic; Pr(> F) = p-value

	Df	Sum of squares	F	Pr (> F)
Co (mg/g)	1	0.4949	14.5266	0.001
Fe (mg/g)	1	0.2048	6.0102	0.006
Pb (mg/g)	1	0.0954	2.7989	0.057
Corg (%)	1	0.1637	4.8052	0.015
TempSe (℃)	1	0.2904	8.5235	0.003
N_total (mg/g)	1	0.0862	2.5317	0.082
NH_4_^+^4 (mg/g)	1	0.0484	1.4204	0.242
NO_3_^+^(mg/g)	1	0.0215	0.6309	0.614
Residual	10	0.3407	NA	NA

### Taxonomic structuring across environmental gradients

Taxonomic composition at the phylum level also exhibited strong site-specific organization (Fig. 3D). The distribution of dominant bacterial groups closely mirrored the geochemical and physical characteristics of each location.

Crater Lake was dominated by Acidobacteriota, Chloroflexota, and Gammatimonadota, forming a distinct assemblage associated with the organic carbon gradient. In contrast, Fumarole Bay exhibited a markedly divergent taxonomic structure, characterized by enrichment in Calditrichota, Planctomycetota, and Patescibacteria, among other phyla restricted to this site, including archaeal phyla such as Nanoarchaeota and Thermoproteota. Meanwhile, Whalers Bay and Livingston clustered along the metal-associated environmental axis and shared a core taxonomic signature dominated by Pseudomonadota, Bacteroidota, and Actinomycetota. Actinomycetota reached its highest relative abundance in Livingston, whereas Pseudomonadota remained a consistent dominant taxon across both sites.

### Co-occurrence of BGCs and ARGs

**Fig. 4:**
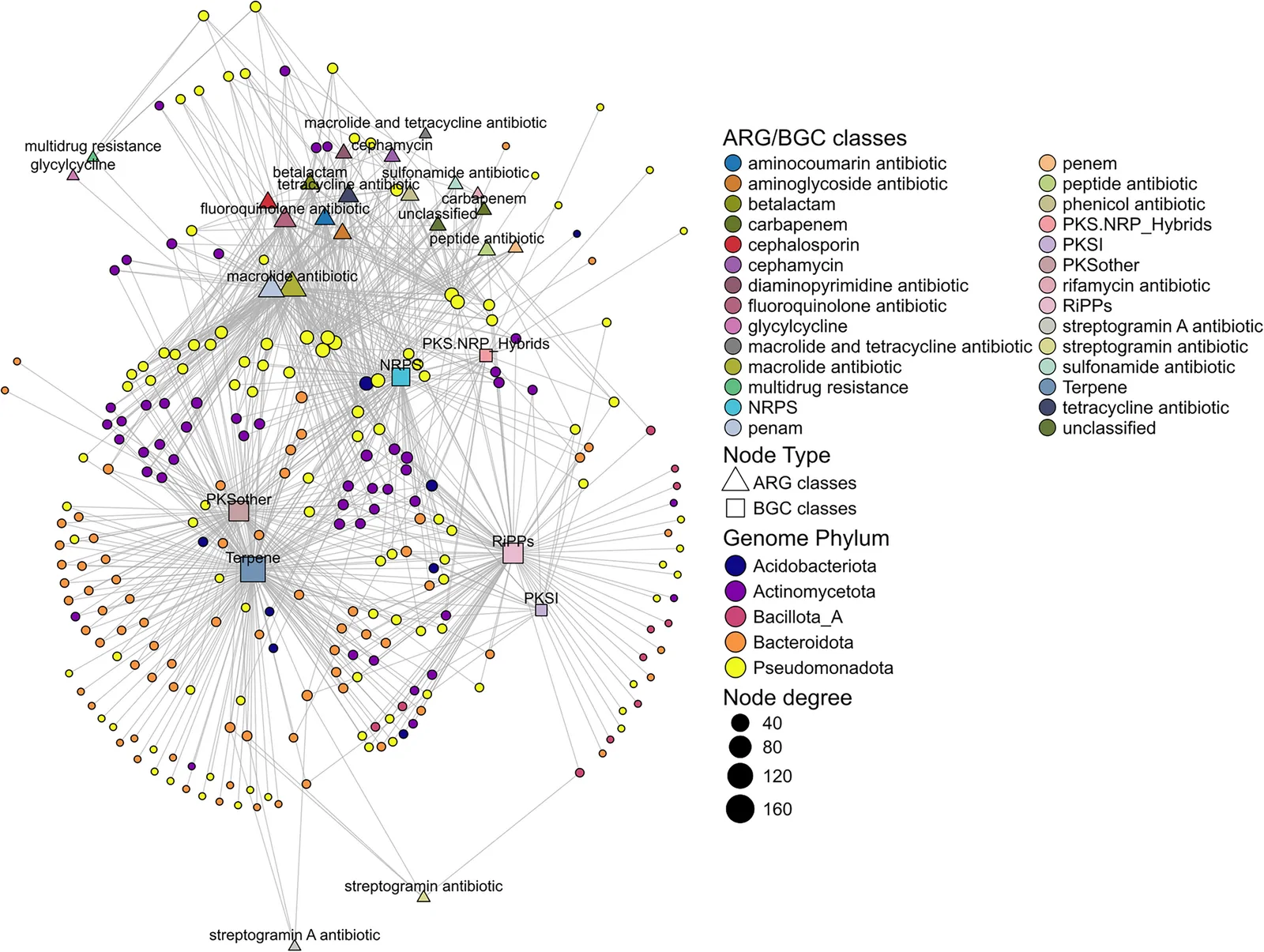
Tripartite MAG–ARG–BGC network from Antarctic metagenomes. Network showing links between MAGs (circles), ARG classes (triangles), and BGC classes (squares). MAGs are colored by phylum, while ARG and BGC nodes are colored by functional class. Node size reflects degree centrality. The Kamada–Kawai layout highlights clusters of biosynthetic and resistance functions and reveals key Antarctic MAGs

To explore the links among microbial taxonomy, antibiotic resistance, and secondary metabolite biosynthesis within the recovered genomes, we constructed a tripartite MAG–ARG–BGC network using a curated annotation dataset. The tripartite network (Fig. 4) revealed a highly structured organization linking ARGs, BGCs, and MAGs.

This representation highlights a clear functional structure across the network. Several ARG classes, including penam and macrolide as major hubs, together with tetracycline, aminoglycoside, and β-lactam, occupied central positions and displayed large node sizes, indicating widespread distribution across MAGs and strong connectivity. In contrast, ARG classes such as streptogramin A, glycycline, and rifamycin were positioned at the periphery, reflecting lower prevalence and weaker structural influence. BGC classes also formed coherent functional communities. NRPS- and PKS-related clusters occupied the upper-central portion of the graph and emerged as biosynthetic hubs. In contrast, RiPPs, Terpene, and PKSI formed distinct modules that preferentially connected to specific MAG groups. At the taxonomic level, MAGs affiliated with Actinomycetota, Pseudomonadota, and Bacillota dominated the central region of the network, showing higher degree values and acting as bridges linking multiple ARG and BGC classes.

To further evaluate the functional linkage between specialized metabolites and the resistome, we investigated the genomic co-occurrence of BGCs and ARGs within individual MAG contigs, yielding a discrete subset of taxa (Table 2). In the phylum Actinomycetota, MAGs LV_1, LV_25, and LV_32 exhibited co-localization of macrolide or aminoglycoside ARGs with polyketide synthase (Type III PKS) or NRPS-like clusters. Notably, MAG LV_32 harbored two distinct NRPS-like clusters, each physically associated with different resistance genes (macrolide and penam). Furthermore, betalactone-type clusters were found co-localized with penam resistance genes in MAGs WB_129 (Actinomycetota) and WB_82 (Bacteroidota). Within the Pseudomonadota, MAG FU_6 contained a thiopeptide (RiPP) cluster associated with an unclassified resistance gene, suggesting a potential link between peptide-based metabolites and specialized defense mechanisms.

**Table 2:** MAGs showing co-localization of BGCs and ARGs within the same cluster

Genome	Family	BGC class	Product Prediction	Resistance
FU_6_Pseudomonadota	Rhodobacteraceae	RiPPs	Thiopeptide	unclassified
LV_1_Actinomycetota	Jatrophihabitantaceae	PKSother	T3PKS	macrolide
LV_25_Actinomycetota	Micrococcaceae	PKSother	T3PKS	aminoglycoside
LV_32_Actinomycetota	Nocardioidaceae	NRPS	NRPS-like	macrolide
		NRPS	NRPS-like	penam
WB_129_Actinomycetota	Micrococcaceae	Others	Betalactone	macrolide
		Others	Betalactone	penam
WB_82_Bacteroidota	Flavobacteriaceae	Others	Betalactone	penam

## Discussion

### Biosynthetic potential in antarctic microbiomes

MAGs provide genome-resolved insights into BGCs and ARGs, particularly in uncultured taxa. Large-scale surveys have shown that MAGs capture extensive taxonomic novelty and reveal previously unseen BGCs, underscoring their role in secondary metabolite discovery (Nayfach et al. 2020; Youngblut et al. 2020). Likewise, MAGs enable host-level assignment of ARGs and their co-localization with mobile genetic elements and virulence factors, improving ecological interpretation of resistomes (Zhao et al. 2020; Abdulkadir et al. 2024). Nonetheless, methodological constraints must be acknowledged, since MAGs are prone to assembly biases, incompleteness, contamination, and strain collapse, which can distort ecological conclusions, with benchmarking studies showing that even high-quality MAGs recover only ~ 77% of core genes and misassign ~ 5% of sequences (Meziti et al. 2021; Yang et al. 2021). Thus, while MAGs provide unique genome-resolved perspectives, their representativeness relative to the entire microbial community is limited, and complementary approaches such as isolate genomes, read-based profiling, or single-cell genomics remain essential for validation (Chen et al. 2019; Pérez-Cobas et al. 2020). In this study, we used MAGs to interrogate the relationships among geochemical gradients, BGCs, and ARGs in Antarctic soils, and our interpretations are necessarily constrained to the MAGs recovered from these sites, recognizing that MAGs do not capture the full microbial diversity present at each study location.

Within these acknowledged limitations, the recovered genomes nonetheless reveal clear patterns of biosynthetic specialization, providing valuable insights into how secondary metabolism contributes to microbial fitness in extreme environments. The dominance of terpene-coding BGCs, which account for over 25% of the total BGC content in our dataset (*n* = 309), supports their proposed ecological role in cold-adaptation mechanisms, such as modulation of membrane fluidity (Avalos et al. 2021; Medeiros et al. 2024). Furthermore, the observed intra-phylum biosynthetic specialization aligns with findings by Belknap et al. (2020), which demonstrated that BGC repertoires can diverge significantly even at the species level. This underscores the necessity of species-level resolution to capture the metabolic landscape of a niche fully. In addition, the geographic shifts in BGC composition (Supplementary Fig. 1) further suggest that local environmental filters actively shape the diversification of biosynthetic pathways. These profiles are likely integral to mitigating extreme stressors, such as oxidative stress and sub-zero temperatures (Bruna et al. 2024; Núñez-Montero et al. 2022; Benaud et al. 2020). Understanding these environmental filters enables a more targeted bioprospecting approach, in which the metabolic response to extreme stress can point to the discovery of novel functional compounds (Medeiros et al. 2025). From a bioprospecting perspective, the scarcity of PKS-NRPS hybrids suggests that these represent specialized biosynthetic innovations, with notable enrichment in certain taxa, such as Calditrichota (Fig. 1A).

The identification of 1,197 BGCs across our datasets reinforces Antarctic soils as reservoirs of unexplored secondary metabolic diversity, and the detection of biosynthetic hotspots, exemplified by the Acidobacteriota MAG *CL_7_Thermoanaerobaculia*, further underscores the untapped potential of largely uncultivated, underexplored Antarctic lineages. Given that natural products remain a major source of therapeutic compounds, the biosynthetic richness of Antarctic microbiomes highlights their dual ecological and biotechnological importance, both as models for understanding microbial adaptation and as sources of novel chemical scaffolds. These estimates, however, should be interpreted cautiously, as short-read sequencing can fragment BGCs across contigs and artificially inflate cluster counts (Waschulin et al. 2021).

NPBDetect analysis predicted significant number of BGCs with antibacterial (*n* = 259) and antifungal (*n* = 73) activities, which aligns with the observed overlap between secondary metabolite production and antibiotic resistance mechanisms, such as efflux pumps and inactivation, as a co-evolutionary relationship between production of antimicrobial compounds and self-protection in these Antarctic niches, further positioning Antarctic environments as a source for new drugs needed to address the global AMR crisis. While these genomic predictions suggest a substantial reservoir of bioactive metabolites, realizing this potential requires overcoming the persistent bottleneck of experimental validation, since many BGCs remain silent under conventional cultivation conditions (Núñez-Montero et al. 2020; Tomm et al. 2019).

### Resistome profiles across sites

The strong spatial segregation of resistome profiles indicates that local environmental conditions play a central role in structuring ARG composition across Antarctic sediments. The high proportion of explained variance attributable to geographic location suggests that deterministic processes, rather than stochastic dispersal, are the primary drivers of resistome distribution in these extreme ecosystems. Similar site-dependent resistome structuring has been reported in Antarctic volcanic sediments and maritime Antarctic soils, where local geochemical conditions emerge as dominant drivers of resistance gene composition (Centurion et al. 2019; Vb et al. 2022).

Whalers Bay exhibits a distinct resistome signature, with selective enrichment of macrolide-, tetracycline-, and rifamycin-resistance determinants, suggesting that localized environmental filtering drives ARG diversification. In sediments from Deception Island, where Whalers Bay is located, Centurion et al. (2019) similarly documented a resistome enriched in both antibiotic- and metal-resistance genes, reinforcing previous evidence that Antarctic volcanic systems can act as resistance hotspots despite minimal direct antibiotic inputs. Environmental modeling (Fig. 3C) further supports this interpretation, as the strong influence of cobalt and iron on resistome structure in this study is consistent with well-established co-selection dynamics between metal resistance genes (MRGs) and ARGs (Baker-Austin et al. 2006).

Heavy metal pressure can maintain antibiotic resistance through co-resistance, cross-resistance, co-regulation, and genetic linkage on mobile elements (Baker-Austin et al. 2006; Gillieatt and Coleman 2024; Zhao et al. 2024). Genomic analyses have shown frequent co-occurrence of ARGs and MRGs within the same plasmids and integrative elements, enabling metal exposure to indirectly select for antibiotic resistance even in the absence of antibiotic pressure (Li et al. 2016; Vats et al. 2022). In Antarctic soils, psychrotolerant bacteria commonly harbor diverse metal-resistance determinants such as *ars*B, *cop*A, *czc*A, and *mer*A, reflecting long-term exposure to metal-driven selection (Romaniuk et al. 2018). Sub-inhibitory metal concentrations can further enhance horizontal gene transfer by activating stress responses and mobilizing mobile genetic elements, facilitating ARG dissemination even in remote environments (Zhang et al. 2018; Gillieatt and Coleman 2024). Consistent with these mechanisms, heavy metal contamination has been associated with co-selection for antibiotic resistance across diverse terrestrial and subsurface environments (Dickinson et al. 2019; Wang et al. 2020). Together, these findings suggest that metal exposure may play an important role in shaping the composition of resistomes in natural microbial communities. In line with this interpretation, the marginal association of the resistome with lead (Pb) observed in this study further indicates a potential legacy effect of historical human activity in Whalers Bay, corroborating previous results from comparative analyses of impacted and non-impacted Antarctic sites showing that Pb and other metals significantly shape resistome profiles (Vb et al. 2022).

Sediment temperature also emerged as a key ecological filter. The thermal anomalies characteristic of Fumarole Bay (varying from 29.9 °C to 48 °C) likely impose strong constraints on community composition, favoring thermotolerant lineages and narrowing resistome diversity to taxa adapted to extreme geochemical conditions. Therefore, environmental stressors, including thermal and metal gradients, can indirectly structure the resistome by selecting microbial taxa that intrinsically harbor broader resistance repertoires (Hao et al., 2020). In contrast, the lack of significant correlations with inorganic nitrogen forms indicates that nutrient enrichment is not the primary determinant of ARG composition across the studied sites. Although penguin-colonized environments such as Hannah Point may exhibit resistome patterns partially influenced by nutrient inputs, previous studies show that metals and microbial community structure explain a greater proportion of the variability in ARG (Zhang et al. 2022). Together, these findings support the role of geochemical stressors, rather than nutrient availability alone, as the dominant drivers of resistome structuring in Antarctic soils.

The parallel structuring observed between taxonomic composition and environmental gradients (Fig. 3D) further supports a site-selection framework that might govern Antarctic microbial communities at the studied sites. Distinct phylum-level assemblages aligned with organic carbon availability, thermal anomalies, and metal-rich conditions indicate that environmental filters shape both microbial identity and functional potential. Mechanistically, metal exposure can structure communities toward taxa enriched in intrinsic resistance genes, thereby indirectly reorganizing ARG pools (Baker-Austin et al. 2006; Hao et al., 2020). Together, these findings suggest that geochemical and physicochemical conditions influence both community composition and the distribution of resistance genes, pointing to potential links between ecosystem structure and the evolution and persistence of the Antarctic resistome.

### Functional implications of BGC-ARG linkage

The network-based framework provides a systems-level perspective on how taxonomic identity, resistance traits, and biosynthetic potential are interconnected within Antarctic microbial communities. This genomic arrangement suggests that antibiotic-producing microbes co-harbor resistance genes to safeguard themselves and neighboring community members against self-produced or co-occurring antimicrobial compounds (Busi et al. 2021). Beyond its structural organization, the network topology provides insights into the ecological strategies of Antarctic microbial communities.

MAGs belonging to Actinomycetota, Pseudomonadota, and Bacillota emerged as key connectors linking resistance and biosynthetic functions. These phyla are widely recognized for their metabolic versatility and stress tolerance, traits that support survival in extreme habitats such as polar environments (Cary et al. 2010; Cavicchioli 2015). Their central position in the network suggests that they may sustain multiple resistance mechanisms while harboring diverse biosynthetic capacities. Simultaneously, in nutrient-limited Antarctic environments, metabolically flexible taxa likely function as reservoirs of adaptive potential. These organisms leverage specialized metabolites to facilitate chemical defense, nutrient acquisition, and complex competitive interactions (Chen et al. 2021).

The pronounced clustering of terpene- and PKS-related BGCs within specific taxonomic groups supports the hypothesis that secondary metabolism is key to adaptive specialization in Antarctic microbial communities and a major contributor to microbial antagonism (Medeiros et al. 2024; O’Brien and Wright 2011). Such antagonistic interactions are fundamental to structuring microbial communities under strong environmental filtering. This metabolic specialization is complemented by the widespread distribution of diverse ARG classes among highly connected MAGs. By modulating competition and promoting coexistence, naturally occurring antimicrobial compounds shape community structure and reinforce ARGs’ role as important contributors to microbial fitness in isolated environments (Allen et al. 2010).

The observed co-localization of BGCs and ARGs within the same genomic regions further underscores a potential functional synergy in which antibiotic production is genetically linked to self-resistance or competitive defense strategies. However, the limited number of detected co-occurrence events likely reflects the inherent technical constraints of short-read sequencing. The fragmented nature of metagenomic assemblies frequently produces truncated contigs that bisect large biosynthetic architectures, hindering the recovery of complete gene neighborhoods (Waschulin et al. 2021). This fragmentation, which can also separate ARGs and mobile genetic elements across contigs, poses a significant challenge for confirming gene linkage by physical proximity. Consequently, the actual frequency of BGC–ARG associations in these environments is likely underestimated. Such assembly fragmentation not only obscures the full metabolic landscape but also limits the detection of genomic signatures of horizontal gene transfer, a primary driver of the dissemination of both biosynthetic genes and resistance genes in extreme environments. Despite these assembly limitations, the hotspots identified in Actinomycetota and Pseudomonadota highlight evolutionary pressure to maintain these defensive and offensive traits in close genomic proximity.

Taken together, integrating network topology with ecological interpretation suggests that a subset of metabolically versatile Antarctic MAGs may represent candidate keystone taxa, linking resistance, biosynthetic potential, and survival strategies. These organisms likely coordinate multiple adaptive traits that enable them to persist in one of Earth’s most physiologically challenging environments.

## Conclusion

This study demonstrates that the Antarctic microbiome is a wealthy reservoir of untapped biosynthetic and resistance diversity, shaped by environmental filtering. By integrating genomic and environmental data, we have shown that secondary metabolism, dominated by terpene, NRPS, and RiPP architectures, is closely associated with microbial adaptation to extreme conditions. The discovery of biosynthetic “hotspots” within underexplored phyla such as Acidobacteriota and Calditrichota, along with their potential antimicrobial activity, underscores these remote environments as critical frontiers for natural product discovery amid the global AMR crisis. Furthermore, the observed co-localization and functional linkage of BGCs and ARGs reveal a sophisticated “biochemical warfare” landscape. The spatial segregation of these functional profiles, particularly the distinct resistome signatures of volcanic sites such as Whalers Bay and Fumarole Bay, indicates that heavy metals and thermal anomalies act as deterministic drivers of resistance evolution. Ultimately, our findings identify metabolically versatile taxa as candidate keystone taxa whose integrated adaptive traits enable persistence in one of Earth’s most extreme habitats. This work provides a systems-level framework linking geochemical stressors to ecosystem structure and specialized metabolism, opening new avenues for targeted bioprospecting in polar regions.

## Supplementary Information

Below is the link to the electronic supplementary material.

Supplementary Material 1 (XLSX 30.5 KB)

Supplementary Material 2 Distribution of BGC Classes within Phyla across sampling locations. Stacked bar charts illustrate the proportional composition of BGC classes across microbial phyla. Each panel represents a distinct sampling location, revealing site-specific variations in the distribution of BGC types. (PNG 419 KB)

## Data Availability

All datasets analyzed in this study are publicly available. The metagenome-assembled genomes are available in the dataset published by Medeiros et al. (2026), with accession numbers provided therein. All scripts and processed data used for analysis are available from the corresponding author upon reasonable request.
